# Digital Light Processing 3D Printing of Isosorbide- and Vanillin-Based Ester and Ester–Imine Thermosets: Structure–Property Recyclability Relationships

**DOI:** 10.1021/acssuschemeng.3c04362

**Published:** 2023-09-19

**Authors:** Anna Liguori, Eugenia Oliva, Marco Sangermano, Minna Hakkarainen

**Affiliations:** † Department of Fibre and Polymer Technology, KTH Royal Institute of Technology, Teknikringen 58, 100 44 Stockholm, Sweden; ‡ Department of Applied Science and Technology, Politecnico di Torino, Corso Duca degli Abruzzi 24, 10129 Torino, Italy

**Keywords:** biobased thermosets, polyester–imines, recycling, digital light processing, 3D printing, photopolymerization

## Abstract

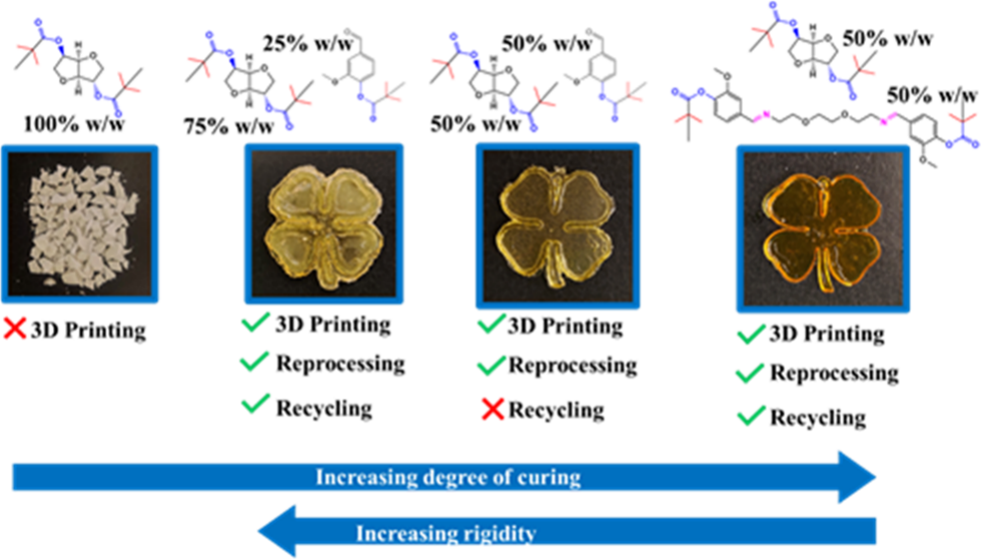

Four isosorbide-based photocurable resins were designed to reveal correlations between the composition and chemical structure, digital light processing (DLP) three-dimensional (3D) printability, thermoset properties, and recyclability. Especially, the role of functional groups, i.e., the concentration of ester groups vs the combination of ester and imine functionalities, in the recyclability of the resins was investigated. The resins consisted of methacrylated isosorbide alone or in combination with methacrylated vanillin or a flexible methacrylated vanillin Schiff-base. The composition of the resins significantly affected their 3D printability as well as the physical and chemical properties of the resulting thermosets. The results indicated the potential of methacrylated isosorbide to confer rigidity to thermosets with some negative effects on the printing quality and solvent-resistance properties. An increase in the methacrylated vanillin concentration in the resin enabled us to overcome these drawbacks, leading, however, to thermosets with lower thermal stability. The replacement of methacrylated vanillin with the methacrylated Schiff-base resin decreased the rigidity of the networks, ensuring, on the other hand, improved solvent-resistance properties. The results highlighted an almost complete preservation of the elastic modulus after the reprocessing or chemical recycling of the ester–imine thermosets, thanks to the presence of two distinct dynamic covalent bonds in the network; however, the concentration of the ester functions in the ester thermosets played a significant role in the success of the chemical recycling procedure.

## Abstract 2

Four isosorbide- and vanillin-based resins and thermosets revealed the relationships between their structure, printability, properties, thermal processability, and chemical recyclability.

## Introduction

Among plant-derived monomers, isosorbide (I), a diol with a bicyclic chemical structure, and vanillin (V), an aromatic monomer with aldehyde, phenolic (−OH), and methoxy groups, are interesting building blocks for the development of rigid biobased thermosets.^1^ Previous studies have documented the potential of the I-molecule for the synthesis of epoxy resins with properties similar to those of bisphenol A diglycidyl ether.^2^ Rigid thermosets with elastic moduli around 2 GPa and thermal stability up to 200 °C were also obtained from the condensation of I with succinic anhydride through a microwave-assisted condensation reaction, followed by polymerization with glycerol.^3^ Thanks to the presence of two hydroxyl functions in its structure, I was also subjected to acrylation and methacrylation reactions and used as the main constituent or reactive diluent in resin formulations. In this context, monoacrylated isosorbide, synthesized from the reaction of I and acrylic acid, was polymerized and treated with succinic anhydride to obtain thermosets with mechanical properties similar to those of commercial epoxy resins.^4^ Thanks to its phenolic nature, vanillin is more competitive than many aliphatic molecules, such as vegetable oils and cellulose, for the development of rigid, hydrophobic solvents and thermally resistant thermosets.^5−8^ Moreover, vanillin can be easily further functionalized.^9−11^

A class of renewable thermosets showing tunable properties was obtained from a combination of methacrylated isosorbide (MI monomer) as a rigid building block and fatty-acid ethyl acrylamide as a flexible component. The resins showed viscosity and curing temperatures suitable for the preparation of composites with *T*_g_ in the range of 136–193 °C obtained by incorporating natural fibers, glass fibers, or carbon fibers into the thermally curable matrix. The matrix turned out to be easily degradable in NaOH solution, thanks to the presence of ester groups in its structure, which was beneficial for the recovery of the reinforcing fibers.^12^ With a similar approach, the MI monomer was copolymerized with acrylated-epoxidized soybean oil, leading to an improvement in the mechanical properties.^13^ Biobased unsaturated polyester thermosets with high thermal and mechanical performances were obtained from a combination of I, 1,4-butanediol, maleic anhydride, and succinic anhydride, with the MI monomer as a reactive diluent. The presence of the MI monomer conferred rigidity to the resulting thermosets (storage modulus in the range of 0.5–3.0 GPa) to overcome the typical solubility drawbacks of biobased diluents as well as the toxicity issues associated with the use of styrene for the curing of unsaturated polyesters.^14^

The presence of ester functions, derived from the methacrylation reaction on the −OH groups of I, endows the resulting thermosets with potential reprocessability. Indeed, ester groups, probably the most commonly used building units in commercial thermosets, belong to the class of dynamic covalent bonds showing also an associative mechanism,^15^ which refers to the possibility of breaking an ester bond and the simultaneous formation of a new one. Therefore, networks with these bonds in their structure are characterized by a vitrimer-like behavior, presenting a constant cross-linking density independent of the reprocessing temperature. However, different from intrinsically reactive, weak, and transient bonds,^16^ triggering the transesterification often requires the introduction of a catalyst in the network,^17,18^ with a possible acceleration of the aging of the ester bonds, which compromises the long-term stability.

Besides the associative mechanism, the ester functions can also follow a dissociative pathway, potentially enabling the degradation of the thermosets at the end of their lifetime. In this context, hydrolysis of the ester bonds in NaOH solutions has been widely reported. As an example, Ma and Webster documented the possibility of fully degrading thermosets obtained by using naturally available dicarboxylic acids to cross-linked epoxidized sucrose soyate in only 13 min.^19^ Using a similar approach, Schen et al. synthesized biobased epoxy thermosets by curing epoxidized vanillic acid, epoxidized plant-based phenolic acids, or epoxidized soybean oil with an anhydride curing agent,^20^ achieving degradation in NaOH solution in duration dependent on the epoxide content, monomer structure, degree of hydrophilicity, cross-linking density, and glass transition temperature. More recently, Le et al. proposed the ring-opening metathesis polymerization of norbornene-functionalized epoxidized soybean oil.^21^ Thanks to the presence of ester bonds, the resulting thermosets were degradable into small oligomers in NaOH or KOH solutions.

The coupling of the hydrolysis and transesterification pathways is considered a plausible mechanism for the degradation of ester thermosets in diol/NaOH catalytic systems. A combination of diols, such as ethylene glycol or poly(ethylene glycol), and a metal salt, such as Zn(OAc)_2_, or an alkaline metal salt, such as NaOH, was reported to favor the degradation of the thermoset,^22^ leading to the obtainment of a mixture of oligomers and diols, which could, in turn, be reused for the obtainment of new thermosets. The limitation of this kind of approach lies in the difficulty in separating the excess diols from the degradation product, which makes the recycling process highly costly and difficult to control.^23^

Among the dynamic covalent bonds, imine functions have been demonstrated to favor the reprocessing and chemical degradation of the thermosets without the need for a catalyst.^24−27^ The reprocessing of imine-based thermosets can take place by exploiting the metathesis pathway, an associative mechanism based on imine exchange.^28,29^ Chemical degradation can occur according to a dissociative mechanism in a slightly acidic aqueous solution, with the consequent reformation of the aldehyde and amine functions, or in the presence of an excess of amines by exploiting the transimination pathway, an associative mechanism intended as an exchange between the imine group and amino functions forming a new imine and a new amine.^28^

In this work, we propose the synthesis of isosorbide- and vanillin-containing thermosets through digital light processing (DLP) three-dimensional (3D) printing of four distinct resin formulations. These resins were designed for systematic structural variations to reveal correlations between the chemical structure, printability, thermoset properties, and especially the recyclability of the thermosets. First, the influence of the ester group concentration was evaluated by designing three thermosets with different amounts of ester groups in their structures. They were obtained from the MI monomer or from a combination of the MI monomer and methacrylated vanillin (MV), a wood-derived monomer widely exploited to obtain aromatic thermosets.^30,31^ Second, the fourth thermoset was obtained by combining the MI monomer and a vanillin-derived Schiff-base resin to evaluate the combination of two dynamic bonds, i.e., ester and imine functions.

This work focuses on the utilization of biobased building blocks (i.e., isosorbide and vanillin) for the design of innovative DLP 3D printable thermosets, aiming especially to understand the relationships between their structure and recyclability. Future work is needed for designing greener processes from monomer synthesis and resin fabrication, with the chemical recycling and purification processes better aligned with the principles of green chemistry.

## Experimental Section

### Materials

Isosorbide(I) (98%), vanillin(V) (99%), methacrylic anhydride (MAA) (≥94%), 4-(dimethylamino)pyridine (DMAP) (≥99%), phenylbis(2,4,6-trimethylbenzoyl)-phosphine oxide (BAPO) (97%), hydrochloric acid (HCl) (37%), and zinc acetate (Zn(OAc)_2_) (99.99%) were purchased from Sigma-Aldrich. 2,2′-(Ethylenedioxy)bis(ethylamine) (Dom) (≥97%) and dichloromethane (DCM) (≥99%) were obtained from Fisher Scientific and sodium bicarbonate (≥99.7%) from Merck. Sodium hydroxide (NaOH) (99%), magnesium sulfate (MgSO_4_) (≥99%), acetone (≥99%), ethanol (EtOH) (100%), and deuterated chloroform (CDCl_3_) (99.8 atom % D) were received from VWR. All chemicals and solvents were used without any additional purification.

### Synthesis of Resins

Methacrylated isosorbide (MI monomer) and methacrylated vanillin (MV monomer) were obtained following procedures similar to those previously reported.^24,25,32,33^ In brief, I (30.00 g, 205.28 mmol) or V (30.00 g, 197.17 mmol) was mixed with MAA (60.60 g, 393.09 mmol for MI; 33.95 g, 220.22 mmol for MV) and DMAP (1.44 g, 11.79 mmol for MI; 0.17 g, 1.39 mmol for MV) in a 250 mL round-bottom flask and kept under stirring at 60 °C for 24 h. The product of each reaction was diluted with DCM and consequently washed with a saturated aqueous solution of sodium bicarbonate, 0.5 M NaOH, 1 M NaOH, and distilled water. The organic phase was dried over MgSO_4_, concentrated at reduced pressure, and dried under vacuum at 30 °C for 2 days, yielding the MI monomer as a white oil (86% reaction yield) and the MV monomer as a white powder (72% reaction yield). The Schiff-base resin (SB monomer) was obtained by subjecting the MV monomer to an imination reaction with Dom, according to the procedure reported in ref (25). In brief, the MV monomer (18.00 g, 81.74 mmol) and Dom (3.65 g, 24.63 mmol) were placed in a 250 mL round-bottom flask and dissolved in 150 mL of DCM. The reaction was carried out at room temperature for 4 h under stirring. Afterward, the reaction mixture was subjected to the same washing and drying procedures as described for methacrylation, yielding the SB monomer as a pale-yellow oil (96% reaction yield).

### Digital Light Processing (DLP) 3D Printing

After the synthesis, the MI monomer alone or in combination with one of the other two monomers was dissolved in DCM with a concentration of 100% w/v (g of resin/mL of DCM) and in the presence of BAPO as a photoinitiator (5% w/w with respect to the total weight of the resin). Four different formulations, according to the compositions reported in Table 1, were prepared and subjected to DLP with a 3D printer (Asiga MAX X27 UV) endowed with a 385 nm light source. Each solution was printed using the exposure times and burn-in exposure times (Table 1) derived from the relative working curves, while the layer thickness and light intensity were set to 0.05 mm and 28.8 mW/cm^2^, respectively, for each solution. After printing, the thermosets were washed in DCM to remove the residual uncured resin and subjected to a UV post-curing step performed in an Asiga Flash UV chamber (385 nm) for 6 min (3 min/side). The thermosets were dried for 2 days in a vacuum oven at 30 °C before being characterized and labeled according to Table 1. Thermosets were printed in the form of rectangular bars (26.00 × 1.53 × 0.75 mm^3^), films (31.71 × 19.77 × 0.75 mm^3^), and clovers (overall area 20.29 × 19.88 × 1.36 mm^3^).

**Table 1:** Abbreviations and Compositions of Different Thermosets and their Printing Parameters

thermoset abbreviations	MI monomer [w/w_tot%_]	MV monomer [w/w_tot%_]	SB monomer [w/w_tot%_]	exposure time for each layer [s]	burn-in exposure time [s]
MI100	100			86	75
MI75	75	25		94	89
MI50	50	50		100	91
SB_MI50	50		50	87	84

### Thermal Reprocessability

The reprocessing was performed by grinding 0.5 g of each thermoset with the help of a mortar and adding 5% (w/w) Zn(OAc)_2_ as a transesterification agent. The resulting powder was transferred to a 25 × 25 × 0.5 mm^3^ square-shaped mold and hot-pressed at 180 °C and 3 MPa for 30 min. All of the thermosets underwent two reprocessing cycles, both performed at the same conditions. Thermosets subjected to one reprocessing were labeled MI75RP1, MI50RP1, and SB_MI50RP1. Thermosets subjected to two reprocessing cycles were labeled MI75RP2, MI50RP2, and SB_MI50RP2.

An additional set of conditions was also employed for the reprocessing of SB_MI50 thermosets, consisting of lowering the hot-press temperature and time to 150 °C and 15 min, respectively, while the applied pressure was kept at 3 MPa. Thermosets reprocessed under these conditions were labeled SB_MI50Mild1 and SB_MI50Mild2, corresponding to one or two reprocessing cycles, respectively.

### Chemical Recycling

MI75 and MI50 were chemically recycled according to the following procedure. 1.2 g of each thermoset was dissolved in 25 mL of 1 M NaOH at 100 °C. The resulting solution was acidified with aqueous HCl (37% w/w) until a pH of 2 and subjected to the evaporation of water at 100 °C. Afterward, ethanol was added, and the mixture was kept under stirring at RT overnight in order to enable the dissolution of the organic molecules and the precipitation of NaCl. The salt was then filtered off, and the resulting solution was dried under reduced pressure. The powder (MI75CR or MI50CR powder) was collected and, after the addition of 5% w/w Zn(AcO)_2_, hot-pressed at 120 °C and 3 MPa for 30 min.

The chemical recycling of SB_MI50 was performed following a previously reported procedure.^17^ 290 mg of the thermosets were dissolved in 6 mL of Dom at 80 °C. The resulting oligomeric product was collected by inducing its precipitation in water, followed by suction filtration. After drying, the amine-end-group-functionalized powder, labeled SBI_MI50CR powder, was mixed with fresh MV monomer with a 3:1 w/w ratio; BAPO and Zn(AcO)_2_ were both added at concentrations of 5% w/w and 2% w/w, respectively. The resulting powder was hot-pressed at 140 °C and 3 MPa for 30 min.

The chemically recycled thermosets were labeled MI75CR, MI50CR, and SB_MI50CR, respectively.

### Characterizations

Chemical structures of the monomers were investigated by ^1^H NMR spectroscopy performed on an Avance 400 (Bruker) spectrometer (400 MHz). CDCl_3_ was used as the solvent and also as the internal standard for calibrating the chemical shift. Chemical structures of all of the monomers as well as the resulting thermosets were characterized using a PerkinElmer Spectrum 2000 Fourier transform infrared (FTIR) spectrometer (Norwalk, CT) equipped with an attenuated total reflectance (ATR) sampling accessory. All of the spectra were recorded in the wavenumber range of 4000–600 cm^–1^ using 16 scans at a resolution of 4 cm^–1^.

The solvent-resistance properties of the thermoset were assessed by immersing each thermoset (around 10 mg) in 2 mL of solvent for 72 h; three common solvents (DCM, EtOH, and acetone) were investigated for the test. After being removed from the solvent, the samples were dried in a vacuum oven at 60 °C until a constant weight was reached. The gel fraction for each sample was determined according to eq 1.
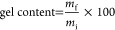
1where *m*_f_ is the final weight of the sample after 72 h of immersion and drying, while *m*_i_ is the initial weight of each sample before the immersion. The analysis was performed in triplicate for each sample and for each solvent.

Thermogravimetry analysis (TGA) of the thermosets and monomers was performed by a TGA/SDTA851e instrument (METTLER TOLEDO). Samples with a weight of around 5 mg were placed in 70 μL ceramic crucibles and subjected to a heating scan from 30 to 600 °C with a heating rate of 10 °C/min and under a 50 mL/min nitrogen flow. Differential scanning calorimetry (DSC) analysis of the thermosets was performed on a METTLER TOLEDO DSC820. Samples with a weight of around 5 mg were sealed into 100 μL aluminum crucibles. All of the samples were first cooled to −10 °C and then subjected to a heating ramp (10 °C/min) up to 200 °C under a nitrogen flow rate of 50 mL/min.

3D printed bars and thermally or chemically reprocessed thermosets were subjected to stress–strain measurements by using an Instron 5944 instrument equipped with a 500 N load cell with a crosshead speed of 0.1 mm/min. Samples were conditioned in a controlled environment with a temperature of 22 °C and 50% relative humidity for 2 days before testing.

## Results and Discussion

**Figure 1:**
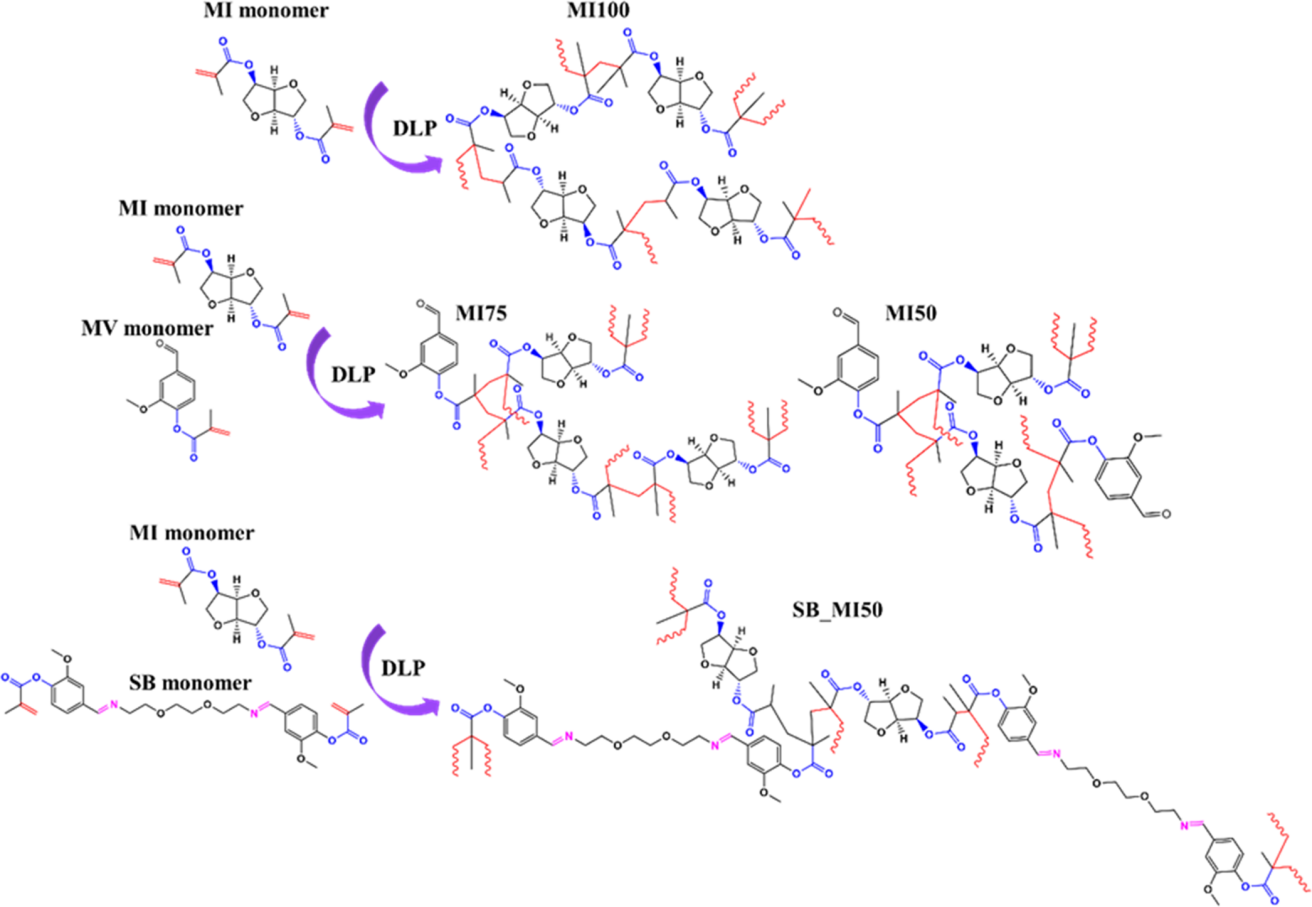
Scheme of the thermosets obtained by the digital light processing 3D printing of single- or bicomponent resins.

Isosorbide- and vanillin-based thermosets with ester or ester/imine functionalities in their structures were obtained through the DLP of methacrylated resins according to the scheme shown in Figure 1. The influence of the rigid and aromatic structure of isosorbide and vanillin, respectively, and the role of the ester and imine dynamic covalent bonds in the properties of the thermosets as well as their thermal reprocessability and chemical recyclability were carefully examined.

### DLP of the Resins

**Figure 2:**
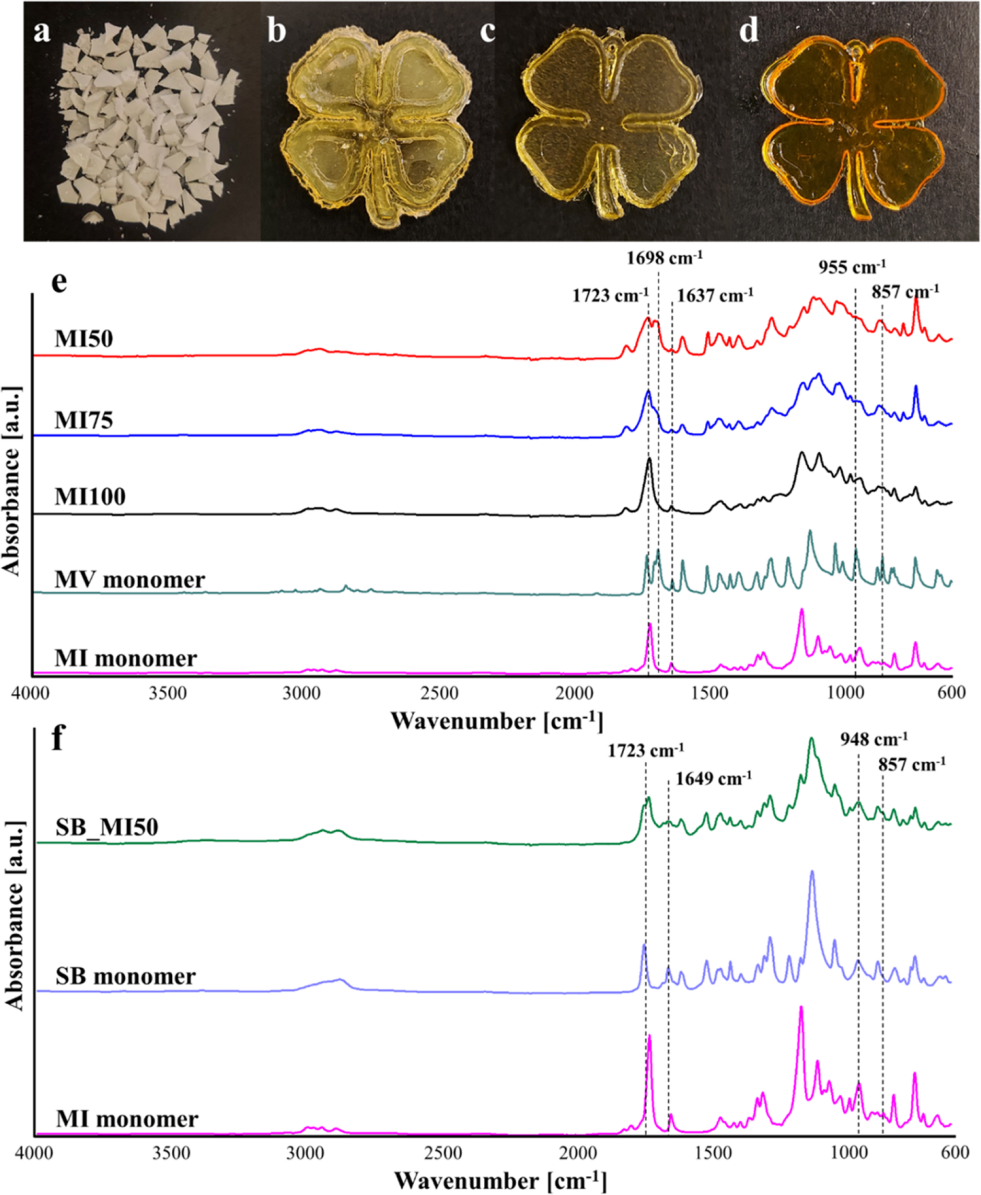
(a–d) Digital light processing 3D printed clovers: (a) MI100, (b) MI75, (c) MI50, and (d) SB_MI50. (e) ATR-FTIR spectra of MI100, MI75, MI50, and their building blocks (MV and MI monomers). (f) ATR-FTIR spectra of SB_MI50 and its building blocks (SB and MI monomers).

First, methacrylation reactions to obtain MI and MV as well as the Schiff-base reaction for the synthesis of the SB monomer were performed, and the reactions were confirmed by the ^1^H NMR analysis reported in Figures S1–S3. Table 1 shows that the different resin formulations and the DLP 3D printing of the formulations clearly correlated with the rigidity of the isosorbide fused bicyclic ring structure.^14,34^ When photopolymerized in the absence of other monomers, the rigidity of the MI monomer might explain the unsuccessful formation of a coherent thermoset. This led to the obtainment of a fragmented MI100 material, as shown in Figure 2a, reflecting the absence of flexible aliphatic segments and the presence of highly packed bicyclic structures (Figure 1). A combination of MI and MV monomers enabled the curing of the resins to continuous and homogeneous thermosets, demonstrating that the MV monomer can act as a spacer among the MI monomeric units, decreasing the density of bicyclic structures and reactive sites and favoring the formation of continuous thermosets (Figure 2b and 2c). The printing fidelity improved with an increase in the MV monomer content, in agreement with the Jacob working curves (Figure S4), which indicate a reduction of the light penetration depths with an increase in the MV monomer concentration in the resins. This result is explained considering the expected higher reactivity of dimethacrylated monomers with respect to monomethacrylated ones.^31^ This facilitates network formation from one side while also inducing a possible overcuring phenomenon. The faster network formation during DLP is also confirmed by the software-determined printing exposure times for the different resin compositions: formulations showing higher contents of MI require shorter exposure times for curing (Table 1). Furthermore, it is also hypothesized that the aromatic aldehydic structure of vanillin might contribute to light absorption, further preventing the propagation of the radiation toward undesired layers of the resin.^35^

The Jacob working curve (Figure S4) demonstrates the greatest propension of the SB–MI resin to prevent overcuring. This is also illustrated by the highest print resolution of the resulting thermoset (Figure 2d). Moreover, the exposure time needed for the curing of this resin turned out to be similar to that recorded for MI100. This agrees with our previous consideration, demonstrating that the presence of dimethacrylated monomers in the formulation improves the reactivity of the resin under UV light, enabling a faster formation of the network. Moreover, in this case, the increased density of aromatic rings and the presence of Schiff-base functions might have a beneficial effect on the prevention of overcuring. Finally, with respect to the MI monomer, the flexibility of the aliphatic segment between the aromatic rings in the SB monomer facilitates the curing of the formulation into a coherent thermoset.

### Characterization of the Thermosets

The ATR-FTIR analysis of MI and MV monomers (Figure 2e) shows in both spectra the presence of absorption peaks at 1723 cm^–1^ ascribable to the carboxyl function of methacrylate esters and at 1637, 950, and 857 cm^–1^ due to the C=C bond stretch of the methacrylate groups.^24,36−38^ Moreover, a peak at 1698 cm^–1^ assigned to the vanillin aldehyde function is detected in the spectrum of the MV monomer. The DLP 3D printing of the resins does not affect the ester groups, which were still present in all three resulting thermosets. Conversely, a noticeable reduction of C=C signals is noted after the printing, confirming the occurrence of photopolymerization. Despite the reduction in intensity, the peak at 1637 cm^–1^ is still detectable, in particular, in MI100, suggesting an incomplete curing of the thermoset. As expected, an increase in the intensity of the aldehyde peak at 1698 cm^–1^ in the thermoset spectra can be observed with an increase in the vanillin content in the formulation. Indeed, while this peak is absent in MI100, it appears as a shoulder of the ester peak in MI75 and as a well-detectable signal in MI50.

The ATR-FTIR spectrum of the SB monomer shows the presence of ester bonds (1723 cm^–1^) and imine functions (1649 cm^–1^), while no signals were detected at 1698 cm^–1^. This agrees with the ^1^H NMR analysis (Figure S3) and confirms the successful imination of the aldehyde functions of vanillin.^25^ Similar to the other thermosets, SB_MI50 preserved the ester functions in its structure as well as the Schiff-base linkages, although this peak partially overlaps with the eventual signal of the C=C stretching vibration at 1637 cm^–1^.

**Figure 3:**
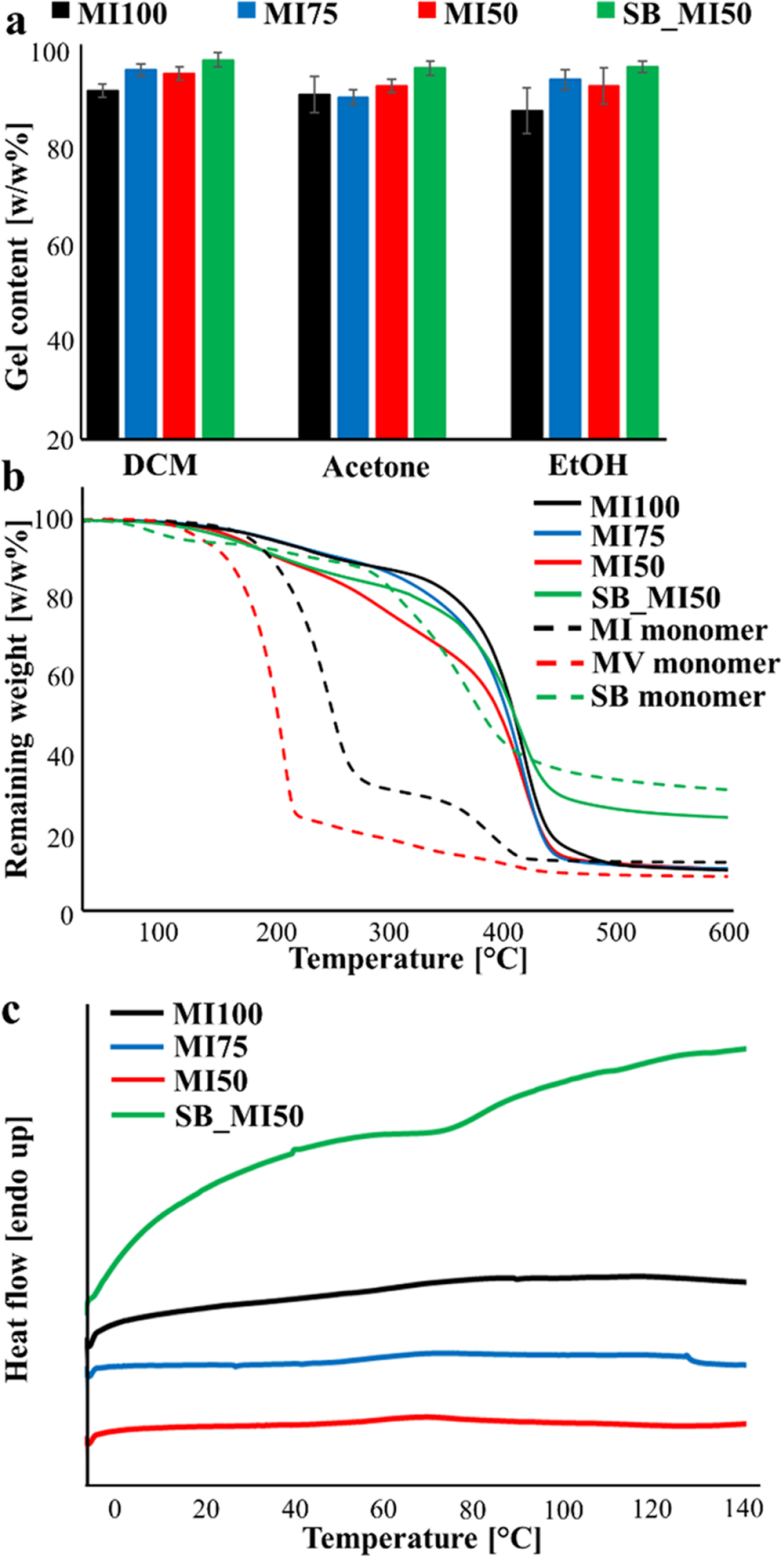
(a) Gel content measurements, (b) TGA curves, and (c) DSC curves of all four thermosets. TGA curves of the monomers are also included.

The gel content measurements, depicted in Figure 3a, clearly highlight the highest solvent-resistance properties of SB_MI50, thanks to the more flexible nature of the SB monomer, which facilitates the curing reaction during DLP. Although the resulting gel contents are always higher than 83% w/w, MI100 shows the poorest solvent-resistance properties among the thermosets, in particular against DCM and EtOH. This behavior is indicative of the lower degree of curing of this thermoset with respect to others, in tune with the ATR-FTIR analysis. The MI monomer has quite a rigid fused bicyclic ring structure, and when one of its two available vinyl groups links to the network through a covalent bond, the mobility of the molecule further decreases and negatively affects the possibility of the unreacted methacrylate finding a radical species to polymerize with.^39^ As a consequence, the curing of the network might remain incomplete, with negative effects on the resulting solvent-resistance properties. The introduction of the MV monomer in the formulation results in beneficial effects on the overall solvent-resistance properties. This might be explained by the higher expected mobility of the MV monomer with respect to the MI monomer. As a consequence, an MV molecule can more easily penetrate through the network acting as a bridge between the unreacted sites of two MI units.

According to the TGA analysis (Figure 3b and Table 2), all thermosets are thermally stable up to around 150 °C and show a similar *T*_deg max_ of around 423 °C. The higher thermal stabilities of MI100 and MI75 in terms of *T*_deg5%_ and *T*_deg30%_ can be ascribed to the higher content of fused bicyclic ring structures.^13,40^ Among the MI–MV thermosets, MI50 shows the lowest thermal stability with a *T*_deg5%_ of around 167 °C and a *T*_deg30%_ of around 337 °C; nonetheless, a higher degree of curing is suggested by the gel content measurements and ATR-FTIR analysis for this network. An explanation for this result lies in the fact that the thermal stability is not only associated with the cross-linking density of the thermosets but it also has a close relationship with the chemical structure of the resins.^13^ Indeed, the replacement of the MI monomer with the MV monomer contributes, on the one hand, to a successful photopolymerization of the resin, but, on the other hand, the presence of aldehyde functions in MV molecules reduces the overall thermal stability as observable from the comparison of the TGA curves of MI and MV monomers (Figure 3b).

**Table 2:** Thermal Properties of the Thermosets

thermoset	*T*_g_ [°C]	*T*_deg5%_ [°C]	*T*_deg30%_ [°C]	*T*_deg max_ [°C]	residue [w/w%]
MI50	60 ± 3	167 ± 4	337 ± 2	423 ± 1	13 ± 2
MI75	56 ± 1	200 ± 2	377 ± 3	423 ± 1	11 ± 3
MI100	64 ± 3	204 ± 5	387 ± 2	424 ± 1	10 ± 2
SB_MI50	93 ± 11	159 ± 7	376 ± 3	422 ± 1	24 ± 2

A comparison of MI50 and SB_MI50 illustrates that the replacement of MV with SB does not induce significant modification of the *T*_deg5%_, while an increase in *T*_deg30%_ was observed, in agreement with the positive contribution of the SB monomer to the thermal stability of the network. The result might be mainly ascribed to the absence of thermally labile aldehyde functions and the collocation of the aromatic rings along the main chains of the network, with a consequent positive effect on the resulting thermoresistant properties. Referring to the previous study in which the thermal stability of an SB thermoset was investigated,^25^ SB_MI50 showed a drastically lower thermal stability with respect to the thermoset photopolymerized from resin consisting only of the SB monomer (*T*_deg5%_: 159 vs 250 °C). This suggests that the partial substitution of a flexible aliphatic resin with rigid bicyclic MI monomers could induce the formation of a network having aromatic–aliphatic segments as branches, which derives from the incomplete polymerization of the SB monomer. From these branches, the depolymerization can start with an overall decrease in the thermal stability of the network. Finally, SB_MI50 shows the highest residue among the considered thermosets in tune with the high residue observed for the SB monomer at 600 °C; this can be explained by considering the higher probability of having adjacent aromatic rings in the network that can more easily lead to the formation of char at high temperatures.

In order to compare the thermosets in terms of *T*_g_ (Figure 3c and Table 2), it is worth considering the fact that this temperature is not only affected by the length of segments between two consecutive cross-links but also highly depends on the chemical structure of these segments as well as the presence of branched units. The quite low and similar *T*_g_ values noted for MI100, MI75, and MI50 are not in tune with the presence of rigid bicyclic units in their structure,^41^ and therefore, the obtained values are likely ascribable to the formation of defective networks during the curing. On the other hand, a high *T*_g_ in the range of 82–104 °C is noted for SB_MI50. This value is significantly higher than that previously noted for the SB thermoset (around 32 °C^25^), indicating the beneficial effect of the MI monomer in increasing the rigidity of the thermoset. Furthermore, the higher *T*_g_ of SB_MI50 with respect to that of MI100 further supports the higher regularity of the bicomponent network and more complete curing reactions. Because of the impossibility of obtaining coherent networks, MI100 was not considered for the investigation of thermal reprocessability and chemical recyclability.

### Thermal Reprocessing and Chemical Recycling

The ester and ester/imine functions present in the considered thermosets belong to the class of associative dynamic covalent bonds and endow the network with potential recyclability. The reprocessing of the ester thermosets, i.e., MI75 and MI50, was performed according to the procedure reported in the Experimental Section. After grinding, the collected fragments were mixed with Zn(AcO)_2_ before being subjected to a hot-press procedure. Zn(AcO)_2_ was selected as the transesterification agent due to its nontoxicity and efficiency.^17,42,43^ In our thermosets, transesterification takes place through the establishment of metal–ion interactions between carboxylate groups and Zn^2+^. The resulting complex acts as a site for ester-exchange reactions,^44,45^ which is targeted to take place during the hot-press step. A similar procedure was employed for the reprocessing of SB_MI50. As already demonstrated in previous studies,^24,25,46−48^ the presence of imine linkages enables the thermal reprocessing of the thermosets according to a metathesis pathway without the need for a catalyst. Considering the presence of ester groups, besides imine functions, Zn(AcO)_2_ was added to the SB_MI50 reprocessing procedure for a more relevant comparison of the reprocessing characteristics of all of the resulting thermosets and the influence of imine bonds on it.

**Figure 4:**
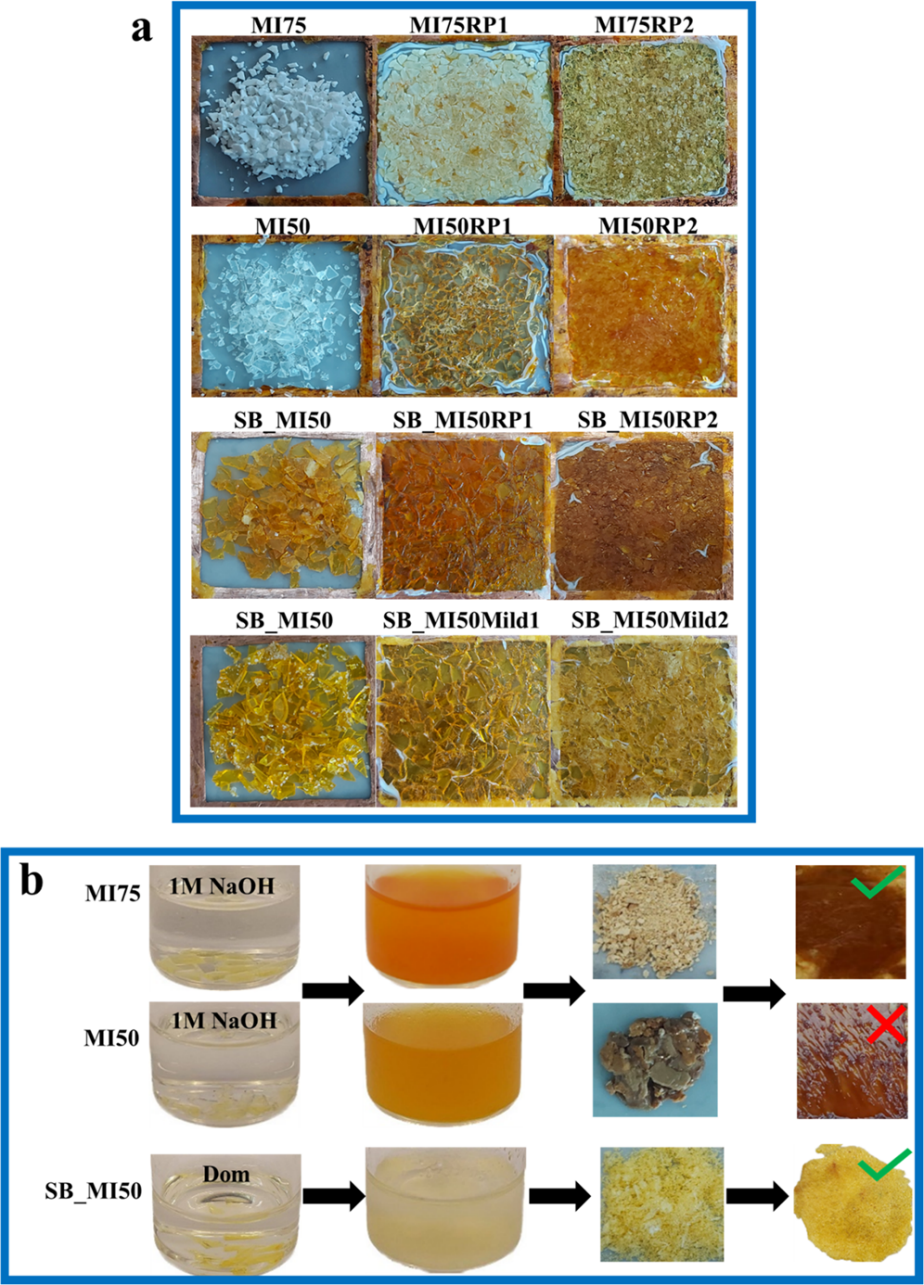
(a) Images of the thermosets after grinding, after the first reprocessing, and after the second reprocessing cycle. For SB_MI50, the images refer to the reprocessing performed under standard conditions (upper row) and under milder conditions (lower row). (b) Scheme of the procedure for the chemical recycling of the thermosets.

As shown in Figure 4a, the films obtained after the second recycling showed higher homogeneity than those subjected to a single reprocessing, probably due to an increased bond exchange reaction with the repetition of the recycling procedure. SB_MI50Mild1 and SB_MI50Mild2 show a paler color with respect to SB_MI50RP1 and SB_MI50RP2, respectively, suggesting that the lowering of the hot-press temperature and time could have beneficial effects on the preservation of the network chemical structure, as further discussed below.

**Figure 5:**
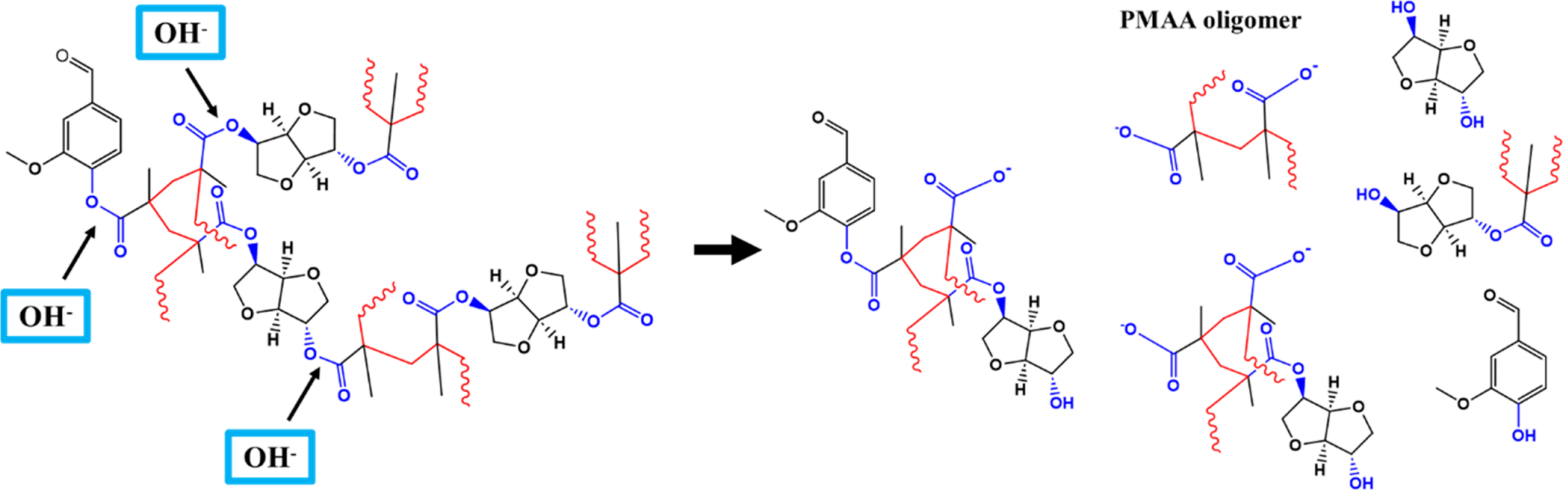
Hydrolysis of ester thermosets in the presence of OH^–^ ions and examples of possible molecules and oligomeric structures produced.

The chemical recycling of the ester thermosets was performed by inducing, as a first step, alkaline hydrolysis of the ester functionalities (Figure 4b, first and second rows) with the consequent opening of the network structure, in agreement with previous studies.^12,23,49,50^ The depolymerization of the network is expected to lead to the obtainment of isosorbide- and vanillin-based oligomeric structures and poly(methacrylic acid) oligomers (PMAA), as schematized in Figure 5. To enable the reformation of the network through the esterification reaction between the isosorbide- and vanillin-based oligomeric structures and PMAA oligomers, the resulting solution was acidified. Indeed, in an alkaline solution, once an ester bond is split into carboxylic acid and alcohol, the hydroxyl anions abstract protons from the acid, leading to the obtainment of negatively charged carboxylate ions with a thermodynamically irreversible process. Conversely, under acidic pH conditions, protons can act as catalysts for esterification, favoring the regeneration of the ester functions between carboxylic acid and −OH groups.^51^ The organic molecules from the recycling, in the form of dried powder, were then recovered through the procedure described in the Experimental Section, mainly consisting of the elimination of the inorganic salt and the evaporation of the solvent.

**Figure 6:**
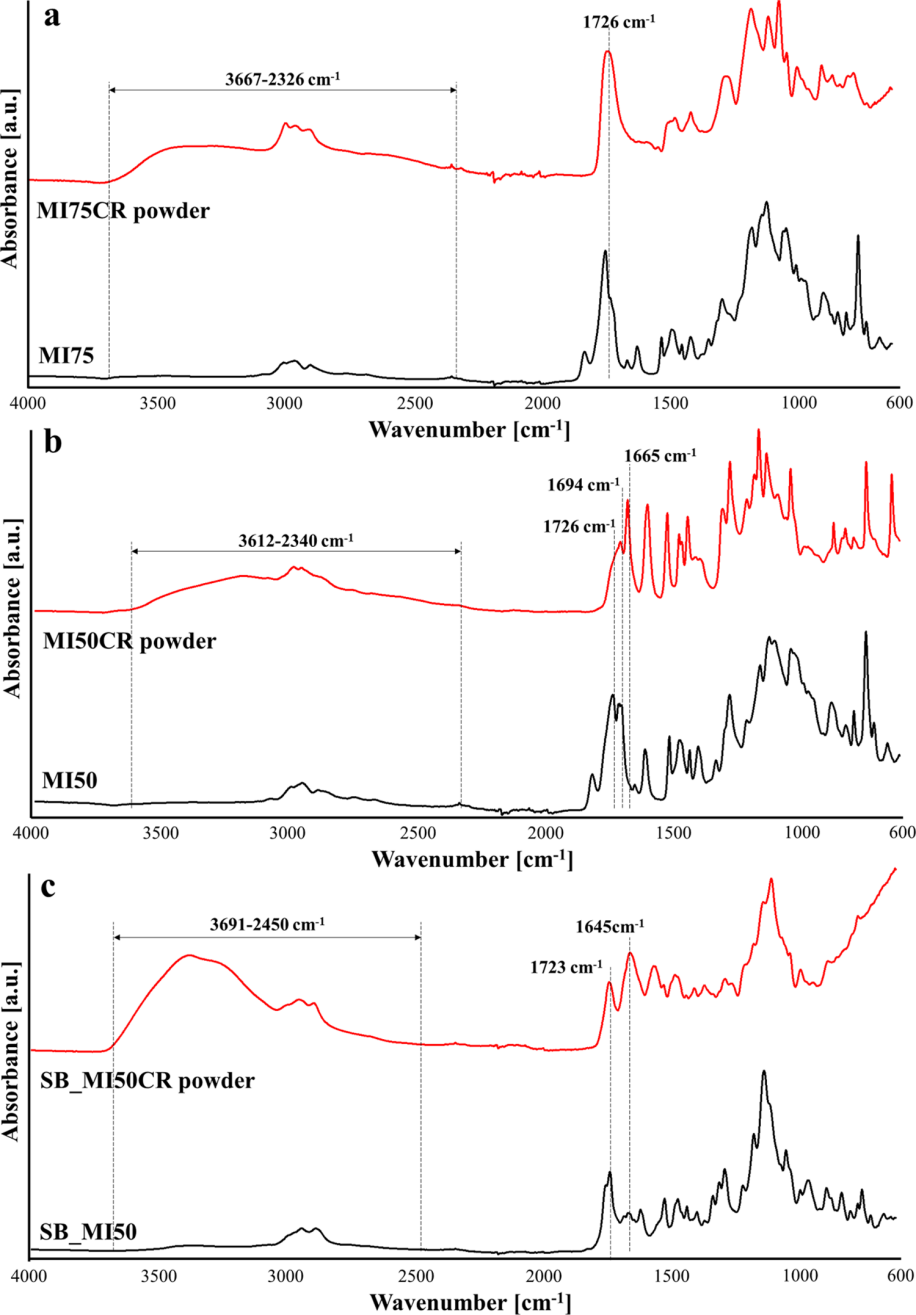
(a) ATR-FTIR spectra of MI75 and MI75CR powders. (b) ATR-FTIR spectra of MI50 and of MI50CR powders. (c) ATR-FTIR spectra of SB_MI50 and SB_MI50CR powders.

The ATR-FTIR spectra of the collected MI75CR and MI50CR powders are displayed in Figure 6a and 6b, respectively. The MI75CR powder clearly shows the presence of −OH groups (3667–2326 cm^–1^) and ester functions (1726 cm^–1^) in its structure, confirming the depolymerization of the network in the form of ester oligomers terminating with −OH groups.^52^ No aldehyde signals are detected in the spectrum of the powder, in tune with the low concentration in the thermosets. Similarly, the MI50CR powder shows the presence of terminal −OH functions; however, the peak of the ester groups is not detected, different from the signal of the aldehydes at 1694 cm^–1^ and the peak at 1665 cm^–1^ typical of both the V and MV monomers. The absence (or low concentration) of ester functions probably highlights the occurrence of almost complete depolymerization of the network in the presence of NaOH with the obtainment of V and I molecules, together with PMAA, as main products. This result could be a consequence of the lower concentration of ester functions in MI50 with respect to MI75, and it can also explain the unsuccessful completion of the chemical recycling procedure of the MI50CR powder. Indeed, while a compact film was obtained after the hot-press step of the MI75CR powder, a jelly and sticky material that was difficult to characterize resulted from the hot-pressing of the MI50CR powder (Figure 4b).

The chemical recycling of SB_MI50 was carried out by inducing the solubilization of the thermoset in Dom, exploiting the transimination pathway of the imine functions.^25^ According to the expected structure, the recovered and dried oligomers (SB_MI50CR powder) showed in their structure the characteristic band of amino groups (3691–2450 cm^–1^) and the characteristic peaks of esters at 1723 cm^–1^ and Schiff-base bonds at 1645 cm^–1^. Considering the intense signal of −NH_2_ functions, aldehydic MV was added together with BAPO and Zn(OAc)_2_ to the powder in order to promote the Schiff-base reaction and the polymerization of MV double bonds directly during the hot-press procedure, favoring the formation of continuous networks.^24^ A continuous film was obtained from the chemical recycling of SB_MI50, as shown in Figure 4b.

**Figure 7:**
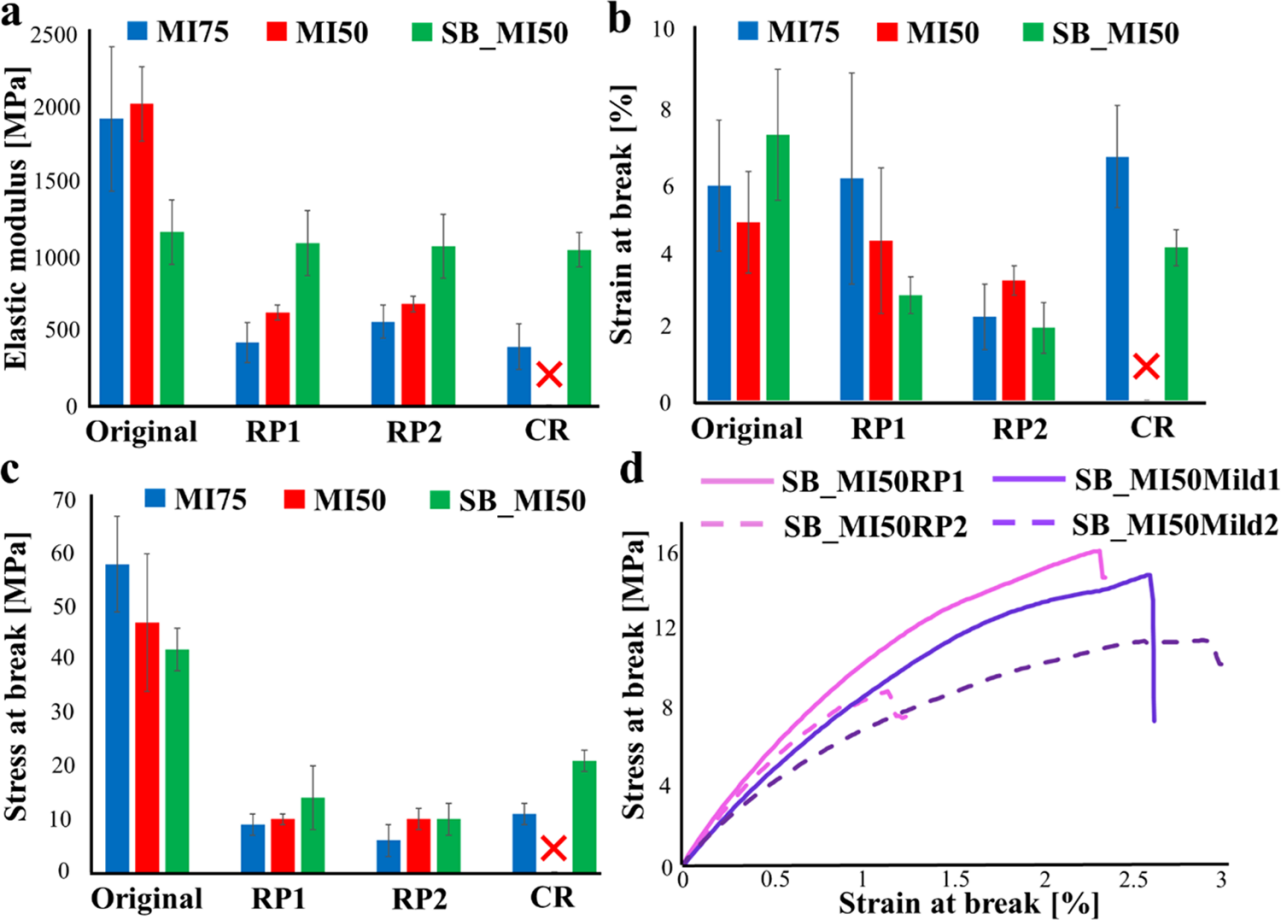
Comparison of the (a) elastic modulus, (b) strain at break, and (c) stress at break among the original thermosets, thermosets after first reprocessing, thermosets after the second reprocessing, and the chemically recycled thermosets. (d) Stress–strain curves of SB-MI50 thermosets after the first and the second reprocessings under standard and milder conditions.

On comparison of their mechanical properties (Figures 7a–c and S5 and Table S1), MI75 and MI50 showed outstanding rigidity (elastic modulus around 2 GPa, stress at break around 50–60 MPa) and resistance to deformation (stress at break around 5%) due to the presence of rigid bicyclic rings of the MI monomer. The replacement of the MV monomer with the SB monomer leads to a reduction of the network rigidity and an increase of strain at break, as expectable considering the introduction of aliphatic segments in the network.

**Figure 8:**
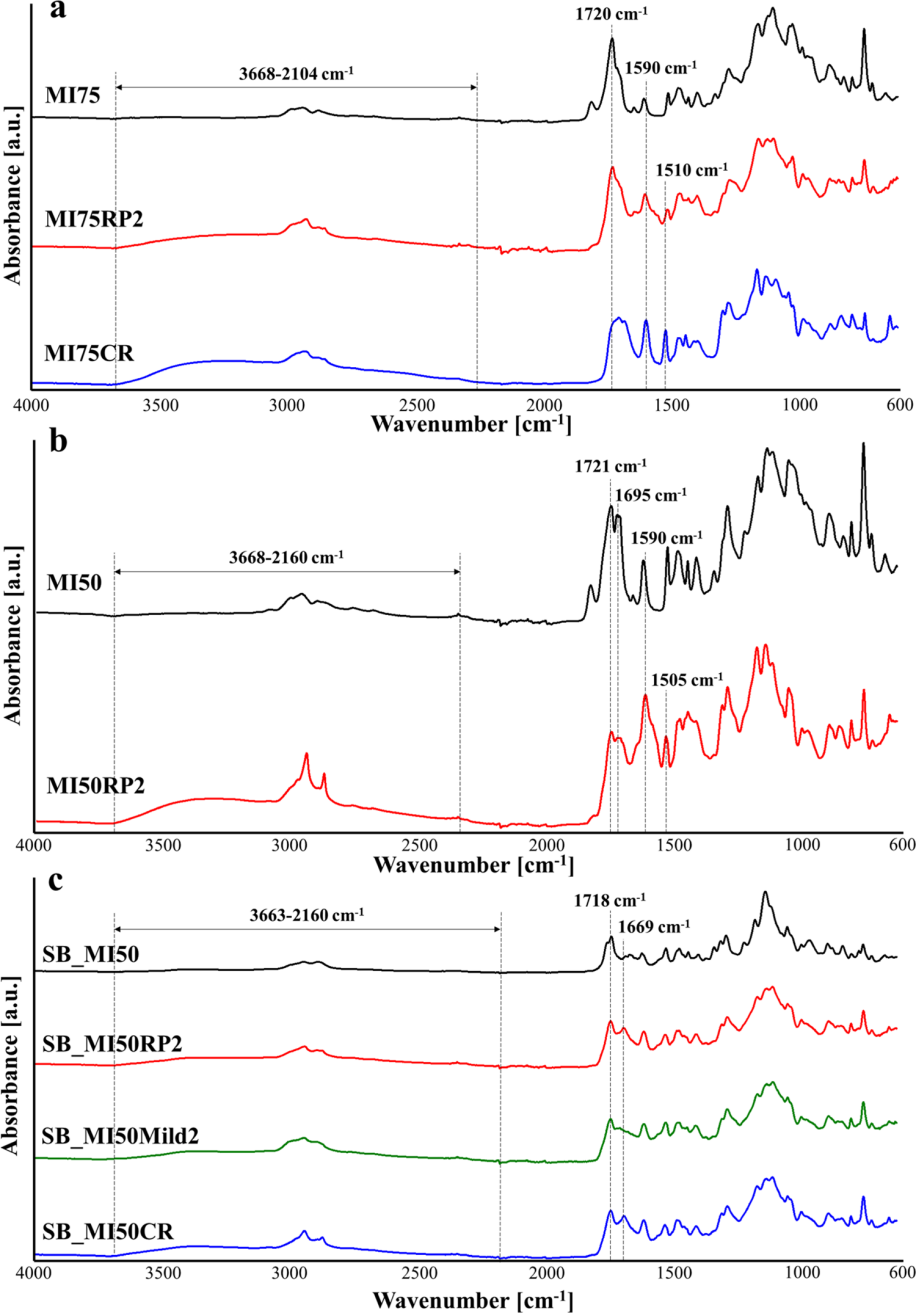
ATR-FTIR spectra of (a) MI75, MI75RP2, and MI75CR; (b) MI50, MI50RP2, and MI50CR; and (c) SB_MI50, SB_MI50RP2, SB_MI50Mild2, and SB_MI50CR.

The reprocessing of ester thermosets induced a drastic deterioration of properties, in particular, in terms of the elastic modulus and stress at break. Besides the damage of nondynamic covalent bonds during the recycling procedure indicated by the modification of the peak at 1590 cm^–1^ and the appearance of a new peak at 1510–1505 cm^–1^ (Figure 8a,8b), the result might also be ascribed to the poor flexibility of the network structures. This hampers the occurrence of transesterification reactions during the hot-press step, with a consequent important reduction of the cross-linking density of the network. In tune with these considerations, a reduction of the strain at break is observed for MI75 with an increase in the number of thermal reprocessing cycles (Figure S6 and Table S1). The ATR-FTIR spectra in Figures 8 further suggest a dissociation of the ester groups during the hot-pressing step, which is particularly evident for MI50. Indeed, after two reprocessing procedures, a relevant decrease in the intensity of the ester peak together with the appearance of an −OH band can be detected for this thermoset. The deterioration of the mechanical properties of MI75 after the chemical recycling might be due to the incomplete esterification reaction between the hydroxyl groups and the carboxylic acids of the oligomers derived from the alkaline hydrolysis in NaOH solution, as indicated by the presence of a high amount of terminal −OH in the recycled network (Figure 8a).

The greater flexibility of the network, together with the simultaneous presence of ester and imine functions, imparts to SB_MI50 reprocessability and chemical recyclability, with practically complete preservation of the elastic modulus. The drop of the strain and stress at break after reprocessing can be ascribed to the damage of covalent bonds that can take place during the grinding, followed by noncomplete restoration during the hot-press step and the opening of imine functions as indicated by the appearance of the aldehyde absorption peak in the SB_MI50MR2 spectrum (Figure 8c). In this context, the reprocessing of SB_MI50 performed under milder conditions did not have significant effects on the mechanical properties of the recycled thermoset (Figure 7d and Table S1), although the reformation of the aldehyde groups was not observed under these conditions (Figure 8c). The results confirmed the sensitivity of the Schiff-base bonds to the hot-press parameters and indicated that the main contributor to the deterioration of the mechanical properties is the damage of nondynamic covalent bonds during the reprocessing procedure. In the case of SB_MI50, the deterioration of the mechanical properties during chemical recycling might derive from the presence of unreacted terminal amines together with the opening of imine functions, as suggested by the shift of the peak of the Schiff-base linkage from 1645 cm^–1^ in the powder (Figure 6c) to 1669 cm^–1^ after the hot-press step (Figure 8c).

Understanding the structural prerequisites for designing more easily recyclable biobased thermosets is important for reaching circular material flows. However, the development of more sustainable materials requires considering all steps of the life cycle of the material. In future work, synthesis procedures need to be developed considering the principles of green chemistry. Here, some fossil-based harmful chemicals and solvents, i.e., MAA and DCM, were used, and the atom economy needs to be improved to limit the production of waste. Some alternative synthetic routes could be developed to improve the atom economy, and MAA could be replaced with biobased alternatives. As an example, Droesbeke and Du Prez utilized the CHEM21 Green Metrics Toolkit to evaluate several synthetic methodologies for methacrylation and acrylation of terpenoids, and the findings of this work could be utilized to develop a greener route for the methacrylation of isosorbide and vanillin.^53^ To increase the biobased content, methacrylic acid and MAA can be obtained through biotechnological fermentation of glucose, leading to methacrylic acid via citramalate;^54^ while MAA can be synthesized from the biobased methacrylic acid, for example, by utilizing di-*tert*-butyldicarbonate in the presence of a catalyst.^55^

Biobased and/or less hazardous solvents have been developed over the years and carefully classified on the basis of their physical properties.^56,57^ Future work is needed to identify suitable alternatives to DCM. During selection, aspects such as density, miscibility with the organic phase, and capacity to solubilize diamines and methacrylated monomers without interfering with the Schiff-base products need to be carefully investigated. An interesting approach to reduce the amount of solvent needed in order to minimize waste might comprise the employment of a microwave-driven approach for monomer synthesis. As an example, Castagnet et al. documented the possibility of using a microwave-assisted procedure combined with enzymatic catalysis for solvent and metal-free acrylation and methacrylation of terpenoid-derived molecules starting with methacrylic acid or methacrylic anhydride.^58^ A similar approach could be evaluated for Schiff-base monomers.

## Conclusions

The design and thorough investigation of four isosorbide-based resins and thermosets revealed relationships between their structure, DLP printability, properties, thermal reprocessability, and chemical recyclability. Isosorbide was methacrylated and subjected to digital light processing 3D printing, but the rigidity of isosorbide prevented the effective curing of the MI100 resin to a coherent thermoset. The MI75 and MI50 resins combining MI and vanillin-derived MV monomers showed better printing quality with an increase in the MV content. Moreover, the better solvent-resistance properties of MI75 and MI50 with respect to MI100 suggested the potential of MV molecules to act as spacers between the rigid MI units. While MI75 and MI100 showed similar thermal stability, this property significantly decreased with increasing MV content (MI50) due to the increased concentration of aldehyde functions. The replacement of the MV monomer with the SB monomer in MI50 led to the obtainment of MI_SB50 thermosets characterized by higher printing fidelity, solvent-resistance properties, and *T*_g_ with respect to all other thermosets. The results indicate a higher degree of curing for this thermoset, ascribable to the presence of a flexible segment in the SB monomer. On the other hand, the flexibility of the SB monomer imparted a decrease in the elastic modulus from around 2 GPa for MI75 and MI50 to 1.2 GPa for SB_MI50.

The presence of ester or ester and imine bonds in the thermosets facilitated reprocessing and chemical recycling. All thermosets were subjected to two reprocessing cycles, and the results indicated a deterioration in the properties of ester thermosets. Conversely, the elastic modulus of SB_MI50 was preserved, which is explained by the contextual presence of both ester and imine functions and the flexibility of the aliphatic segments in the network. Moreover, the investigation of the effects of the hot-press conditions, in particular temperature and time, on the recyclability of SB_MI50 indicated that a decrease from 180 and 30 min to 150 and 15 min, respectively, did not influence the resulting mechanical properties, while it enabled a better preservation of the network chemical structure, preventing the opening of the Schiff-base bonds.

Ester thermosets were chemically recycled by exploiting as a first step the hydrolysis of ester functions in NaOH solutions, followed by the reformation of the ester groups during the hot-pressing. While MI75 could be chemically recycled through this process, the lower concentration of ester groups in MI50 prevented successful chemical recycling. However, even for MI75, there was a drastic drop in the elastic modulus, probably due to the incomplete reformation of the ester functions. The ester–imine thermoset, MI_SB50, was chemically recycled by dissolving it in an excess of diamine to utilize the transimination pathway. The resulting oligomeric structures showing terminal amines were then hot-pressed in the presence of the virgin MV monomer in order to induce the reformation of the network. Similar to the reprocessed thermosets, in this case, the elastic modulus of MI_SB50 was preserved after the chemical recycling procedure. Our results pave the way for more circular isosorbide-based resins and thermosets. At the same time, increasing the sustainability of the resin fabrication and the chemical recycling processes, including purification steps, are topics that should be explored in future work.

## Supporting Information Available

^1^H NMR spectra of the MI, MV, and SB monomers. Working curves for the printing of the different resins. Stress–strain curves of the printed thermosets, curves of MI75MR1, MI75MR2, and MI75CR, and those of MI50MR1 and MI50MR2. Mechanical properties of all printed, reprocessed, and chemically recycled thermosets (PDF)
