# Supplementary material for: Digital Light Processing 3D Printing of Isosorbide- and Vanillin-Based Ester and Ester–Imine Thermosets: Structure–Property Recyclability Relationships
Source: ACS Sustain Chem Eng. 2023 Sep 19;11(39):14601–13. doi: 10.1021/acssuschemeng.3c04362 (PMC10548585; doi:10.1021/acssuschemeng.3c04362)
Supplement: Supplementary file 1 — sc3c04362_si_001.pdf [file sc3c04362_si_001.pdf]

Supplementary Material

Digital light processing 3D printing of isosorbide- and vanillin-based ester and ester-imine thermosets: Structure-property-recyclability relationships

Anna Liguori [a], Eugenia Oliva [a,b], Marco Sangermano [b], and Minna Hakkarainen [a,*]

[a] KTH Royal Institute of Technology, Department of Fibre and Polymer Technology, Teknikringen 58, 100 44 Stockholm, Sweden

[b] Politecnico di Torino, Department of Applied Science and Technology, Corso Duca degli Abruzzi 24, 10129 Torino, Italy

This supporting information file contains 7 Figures and 1 Table on 5 pages.

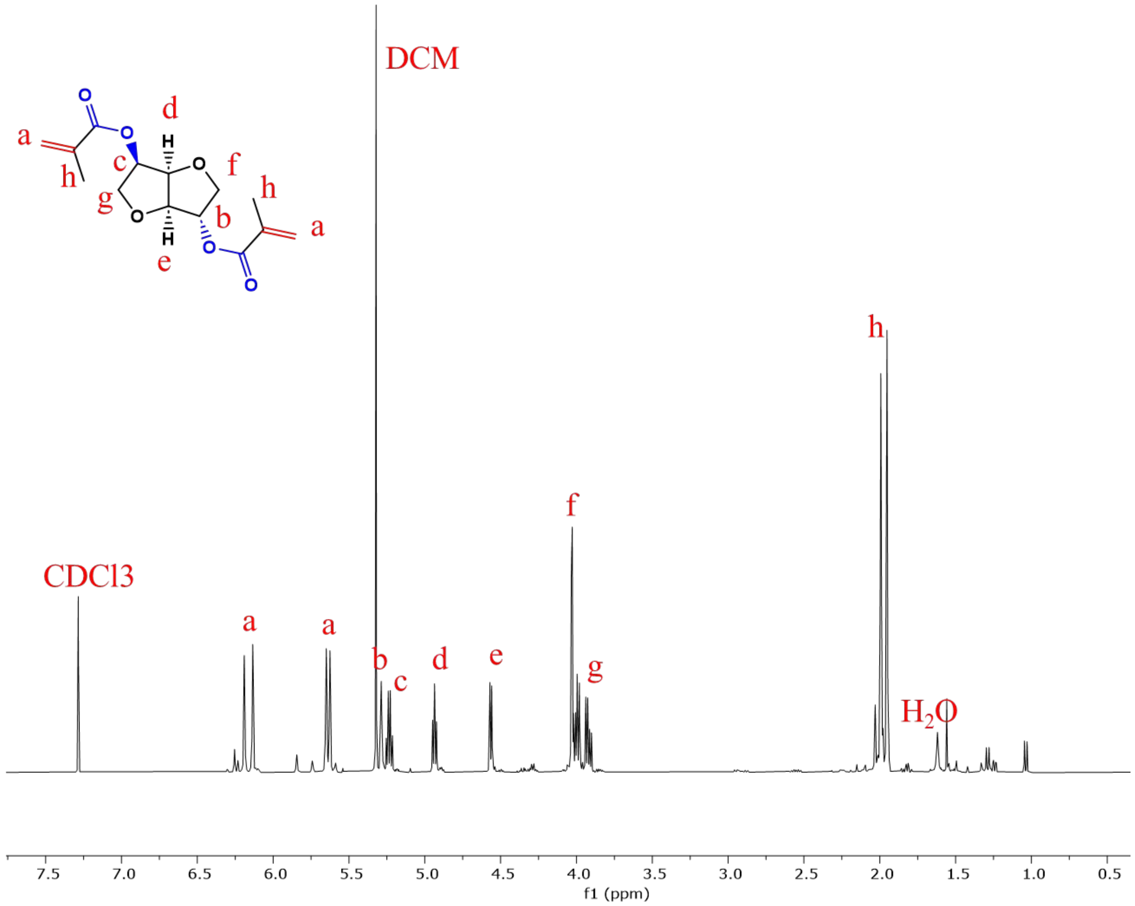

Figure S1. ^{1}H NMR spectrum of MI monomer.

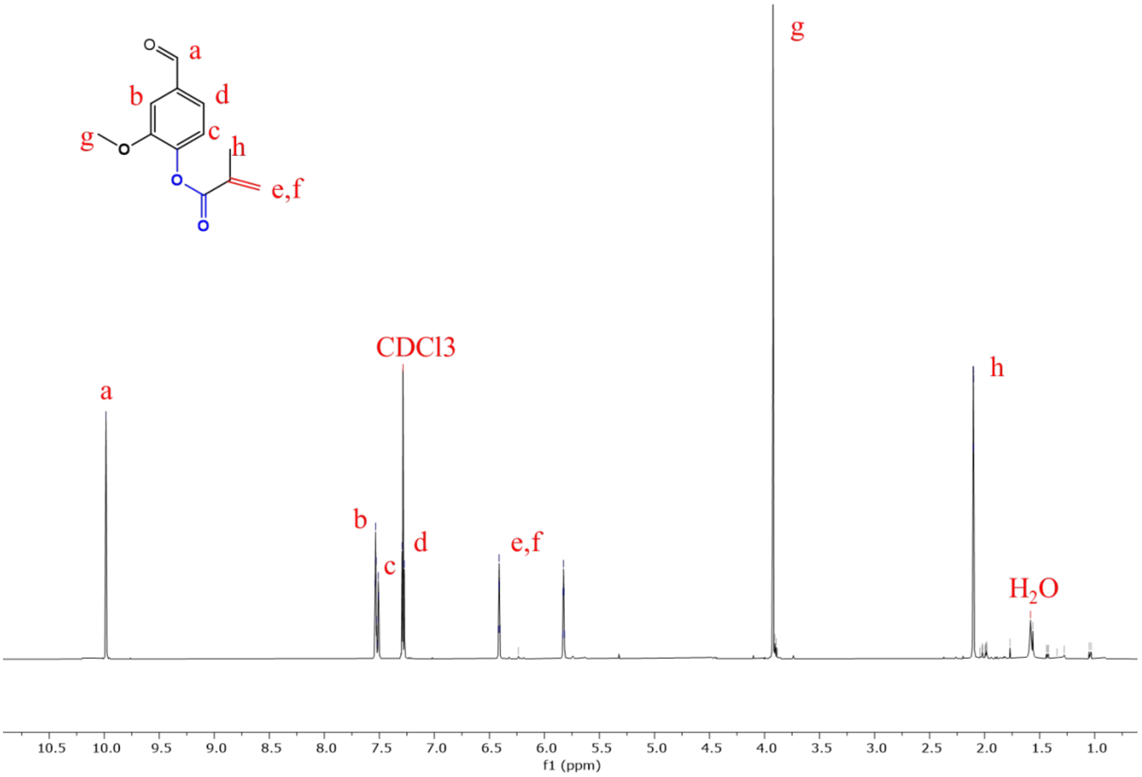

Figure S2. ^{1}H NMR spectrum of MV monomer.

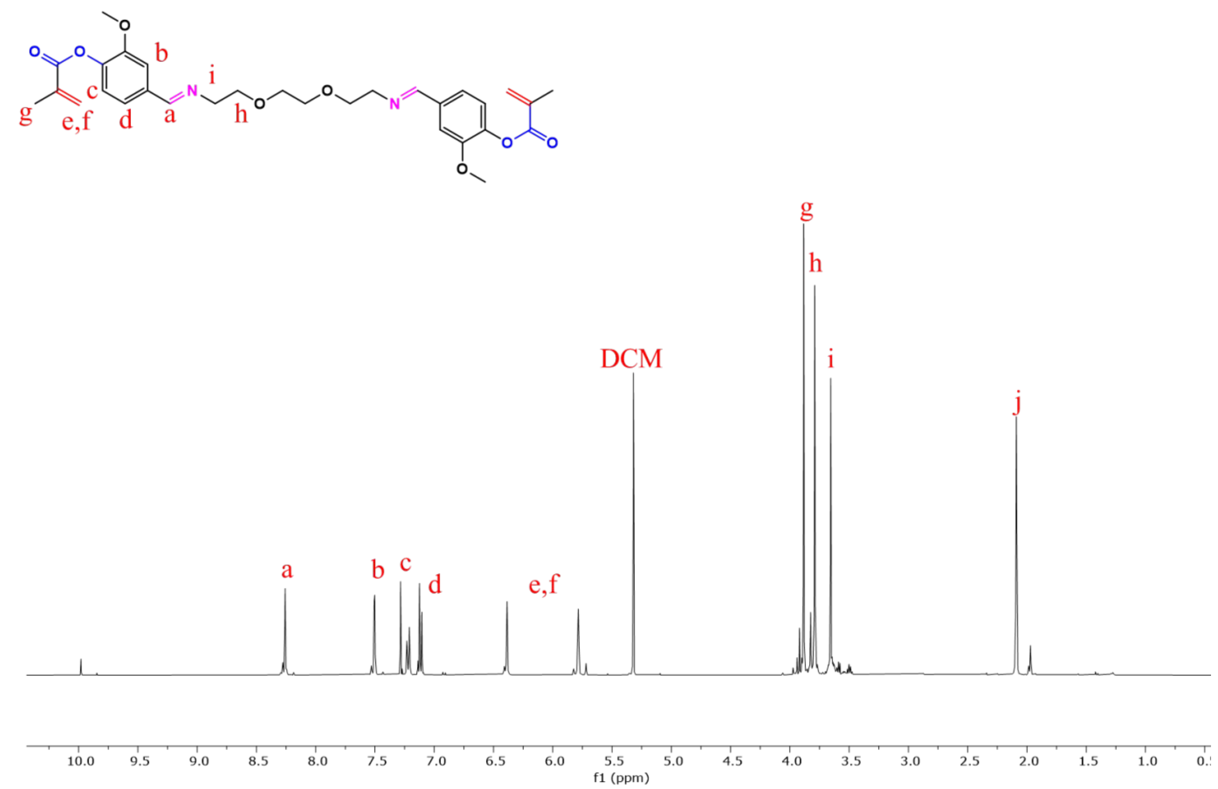

Figure S3. ^{1}H NMR spectrum of SB monomer.

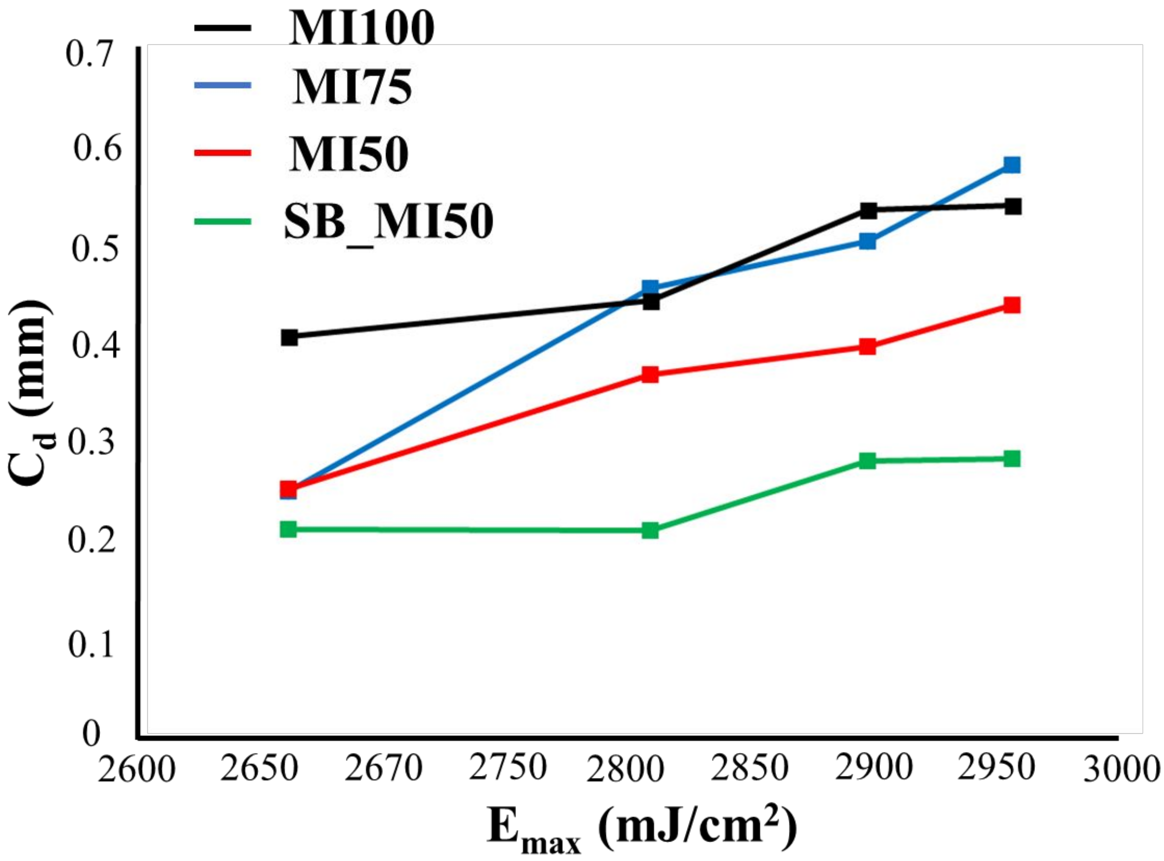

Figure S4. Working curves for printing of the different resins.

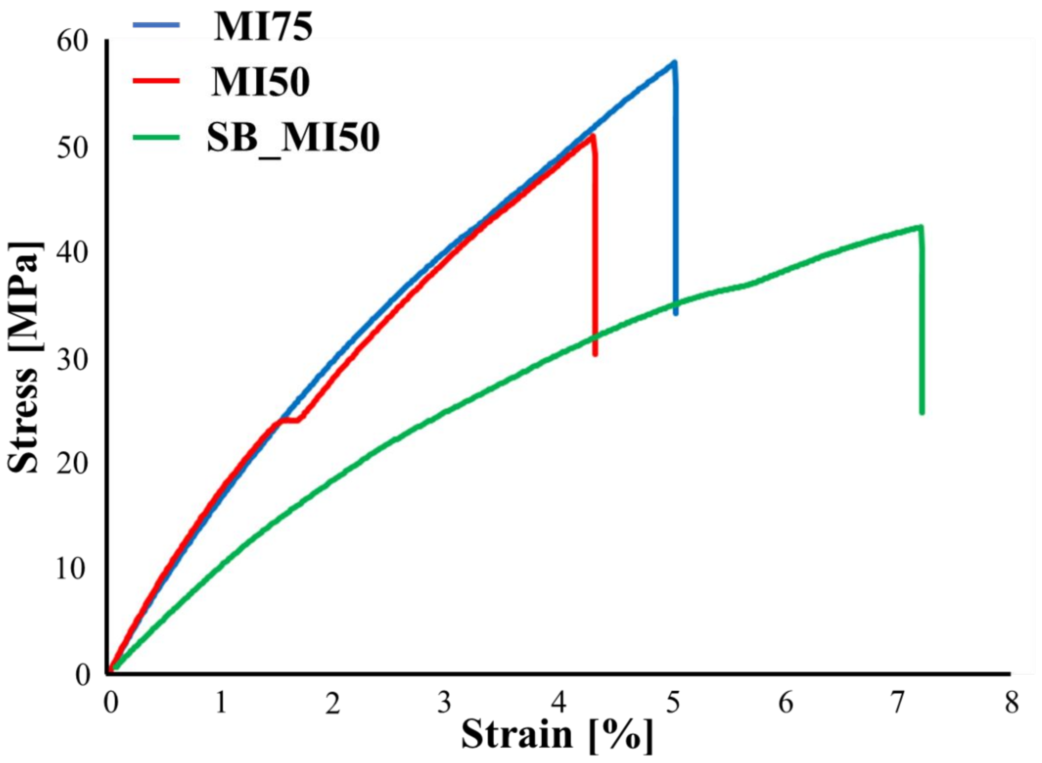

Figure S5. Stress-strain curves of the printed thermosets.

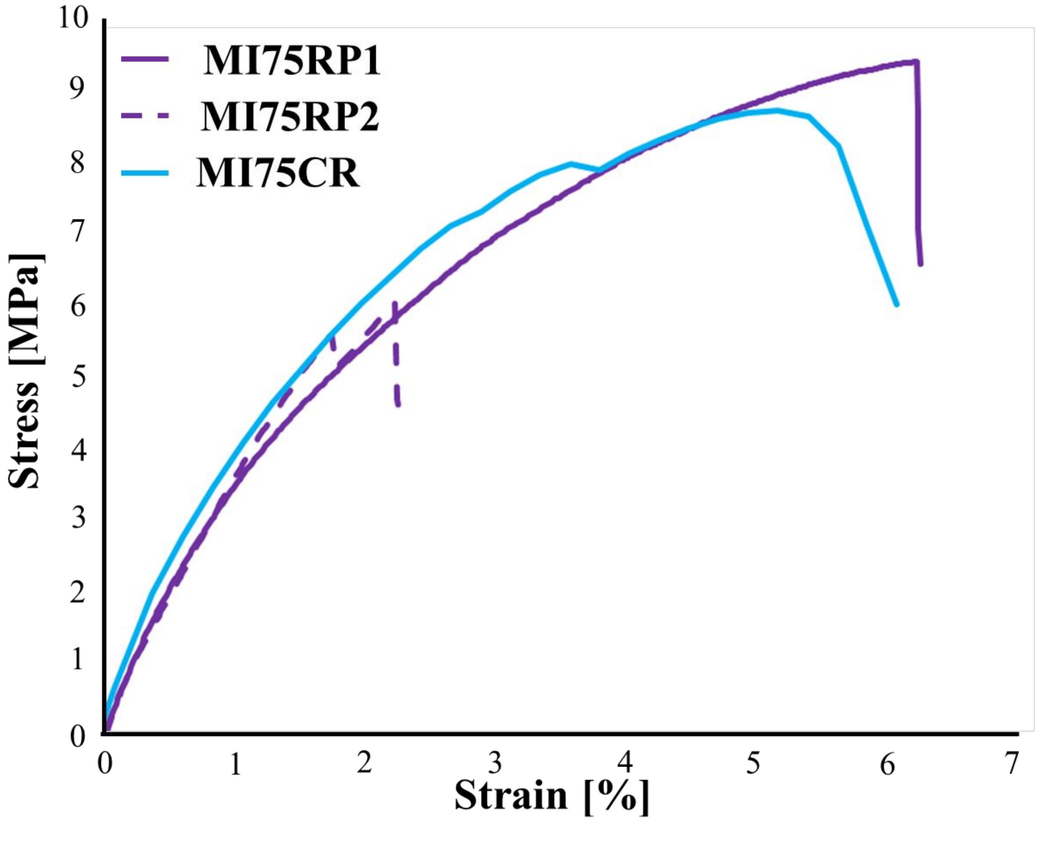

Figure S6. Stress-strain curves of MI75RP1, MI75RP2 and MI75CR.

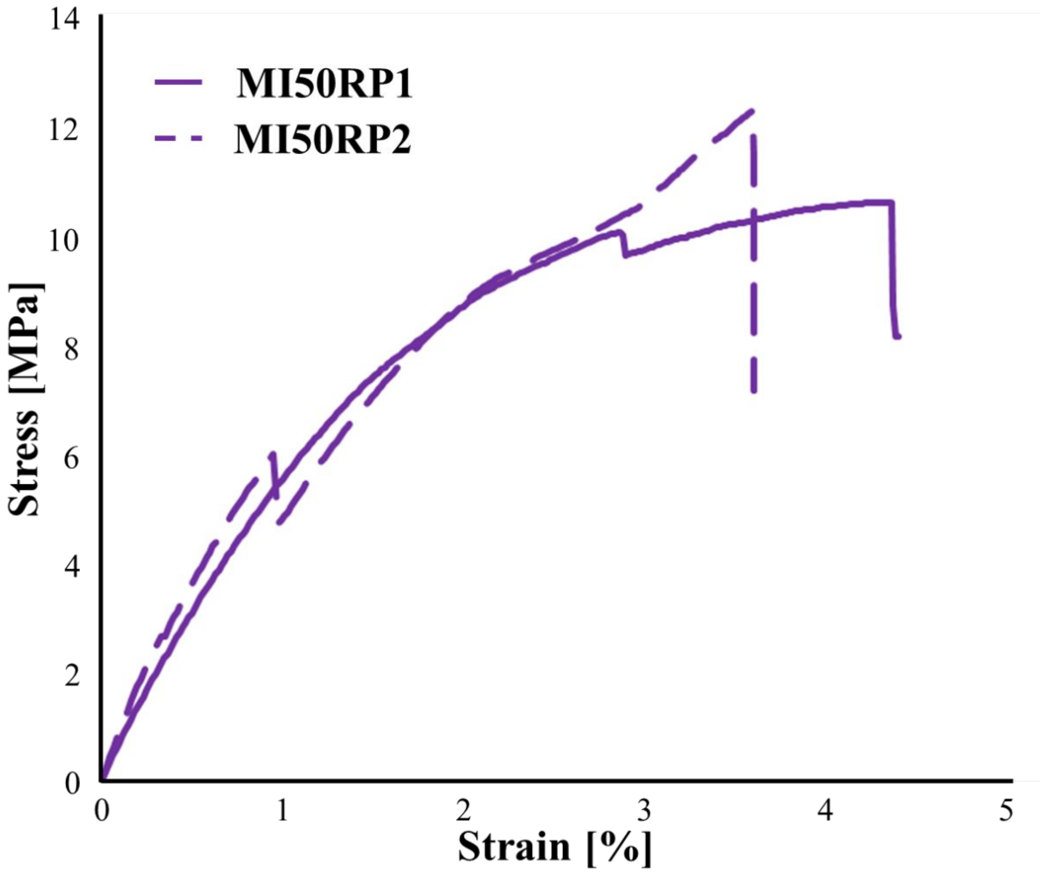

Figure S7. Stress-strain curves of MI50RP1 and MI50RP2.

Table S1. Stress strain measurements.

Sample	Elastic modulus [MPa]	Stress at break [MPa]	Elongation at break [%]
**MI50**	2026 ± 250	47 ± 13	5 ± 1
**MI50RP1**	624 ± 51	10 ± 1	4 ± 2
**MI50RP2**	681 ± 52	10 ± 2	3 ± 1
**MI75**	1925 ± 484	58 ± 9	6 ± 2
**MI75RP1**	421 ± 134	9 ± 2	6 ± 3
**MI75RP2**	562 ± 110	6 ± 3	2 ± 1
**MI75CR**	393 ± 152	11 ± 2	7 ± 1
**SB_MI50**	1163 ± 215	42 ± 4	7 ± 2
**SB_MI50RP1**	1088 ± 217	14 ± 6	3 ± 1
**SB_MI50RP2**	1070 ± 214	10 ± 3	2 ± 1
**SB_MI50Mild1**	1229 ± 271	15 ± 4	2 ± 0
**SB_MI50Mild2**	876 ± 62	9 ± 2	3 ± 0
**SB_MI50CR**	1045 ± 116	21 ± 2	4 ± 1
